# SNX32 is a host restriction factor that degrades African swine fever virus CP204L via the RAB1B-dependent autophagy pathway

**DOI:** 10.1128/jvi.01599-23

**Published:** 2024-01-03

**Authors:** Wenping Yang, Lingxia Li, Jing Zhang, Junhuang Wu, Weifang Kang, Yue Wang, Haiyan Ding, Dan Li, Haixue Zheng

**Affiliations:** 1 State Key Laboratory for Animal Disease Control and Prevention, College of Veterinary Medicine, Lanzhou University, Lanzhou Veterinary Research Institute, Chinese Academy of Agricultural Sciences, Lanzhou, China; 2 Gansu Province Research Center for Basic Disciplines of Pathogen Biology, Lanzhou, China; Lerner Research Institute, Cleveland Clinic, Cleveland, Ohio, USA

**Keywords:** ASFV, CP204L, SNX32, RAB1B, autophagic degradation

## Abstract

African swine fever virus (ASFV) causes a highly contagious and deadly disease in domestic pigs and European wild boars, posing a severe threat to the global pig industry. ASFV CP204L, a highly immunogenic protein, is produced during the early stages of ASFV infection. However, the impact of CP204L protein-interacting partners on the outcome of ASFV infection is poorly understood. To accomplish this, coimmunoprecipitation and mass spectrometry analysis were conducted in ASFV-infected porcine alveolar macrophages (PAMs). We have demonstrated that sorting nexin 32 (SNX32) is a CP204L-binding protein and that CP204L interacted and colocalized with SNX32 in ASFV-infected PAMs. ASFV growth and replication were promoted by silencing SNX32 and suppressed by overexpressing SNX32. SNX32 degraded CP204L by recruiting the autophagy-related protein Ras-related protein Rab-1b (RAB1B). RAB1B overexpression inhibited ASFV replication, while knockdown of RAB1B had the opposite effect. Additionally, RAB1B, SNX32, and CP204L formed a complex upon ASFV infection. Taken together, this study demonstrates that SNX32 antagonizes ASFV growth and replication by recruiting the autophagy-related protein RAB1B. This finding extends our understanding of the interaction between ASFV CP204L and its host and provides new insights into exploring the relationship between ASFV infection and autophagy.

**IMPORTANCE:**

African swine fever (ASF) is a highly contagious and acute hemorrhagic viral disease with a high mortality near 100% in domestic pigs. ASF virus (ASFV), which is the only member of the family *Asfarviridae*, is a dsDNA virus of great complexity and size, encoding more than 150 proteins. Currently, there are no available vaccines against ASFV. ASFV CP204L represents the most abundantly expressed viral protein early in infection and plays an important role in regulating ASFV replication. However, the mechanism by which the interaction between ASFV CP204L and host proteins affects ASFV replication remains unclear. In this study, we demonstrated that the cellular protein SNX32 interacted with CP204L and degraded CP204L by upregulating the autophagy-related protein RAB1B. In summary, this study will help us understand the interaction mechanism between CP204L and its host upon infection and provide new insights for the development of vaccines and antiviral drugs.

## INTRODUCTION

African swine fever (ASF) is a highly contagious viral disease of domestic and wild pigs, with a mortality rate of almost 100% (1–4). No commercial vaccines or antiviral treatments are available for the prevention and control of the disease (5–8). Outbreaks of ASF have caused huge economic losses to the pig industry worldwide. African swine fever virus (ASFV), which belongs to the family *Asfarviridae*, is the etiological agent of ASF. ASFV is a large, enveloped virus with icosahedral morphology and an average diameter of 200 nm (9). The ASFV genome is a linear double-stranded DNA molecule of 170 to 190 kb that encodes between 151 and 167 open reading frames, depending on the virus strain (10, 11).

Among ASFV structural proteins, the p30 protein is encoded by the viral gene CP204L and is abundantly expressed in the early stages of infection (12, 13). CP204L is a membrane-localized and highly antigenic protein with phosphorylation sites (14, 15). Despite its significance in virus infection, the interaction between CP204L and host proteins is relatively unknown. It has been reported that cellular protein heterogeneous nuclear ribonucleoprotein K interacts with CP204L and downregulates host cell mRNA translation (16). Cellular homotypic fusion and protein sorting (HOPS) protein VPS39 interacts with CP204L and participates in the synthesis of viral proteins and virus replication (17). There is also a report that CP204L is related to endocytosis, actin cytoskeleton regulation, and innate immunity through a membrane yeast two-hybrid system (18). Previous studies have shown that CP204L is essential for ASFV replication, is involved in ASFV entry, and could elicit virus-neutralizing antibodies in infected animals (2, 19, 20). However, the detailed mechanism remains unknown. Thus, deciphering the mechanism regulating the interaction between CP204L and the host remains of great interest and importance in controlling ASF.

Sorting nexins (SNX) are a large group of diverse cellular trafficking proteins, with 33 members identified in mammals (21). Based on their scaffolding, enzymatic, and regulatory domains, the members are divided into different subfamilies (22). Most of them share a common phox homology domain. Many SNX family members also contain various other conserved structural domains (21, 23, 24) and are closely related to the regulation of virus replication. SNX5 is essential for virus-induced autophagy, but not for basal or stress- or endosome-induced autophagy. PI3KC3-C1 activation at endosomes depends on SNX5 for initiating autophagy during viral infection (25); SNX27, together with the retromer complex, prevents the ACE2/virus complex from entering lysosomes/late endosomes, decreasing viral entry in cells where the endocytic pathway dominates (26). Human respiratory syncytial virus (HRSV) N and M proteins interact with SNX2, which is important in the traffic of HRSV structural proteins toward assembly sites (27). The interaction between SNX5 and UL35 proteins is required for efficient viral replication and transport of the most abundant human cytomegalovirus glycoprotein B (28). At present, it is still unclear whether the SNX protein is involved in regulating ASFV replication.

In this study, we screened and confirmed that the cellular protein SNX32 interacted with and degraded CP204L by upregulating the autophagy-associated molecule RAB1B. Overall, our study reveals a new mechanism of the host protein defense against ASFV infection, which may provide novel strategies for the development of antiviral drugs and vaccines.

## RESULTS

### ASFV CP204L interacts with SNX32

**Fig 1:**
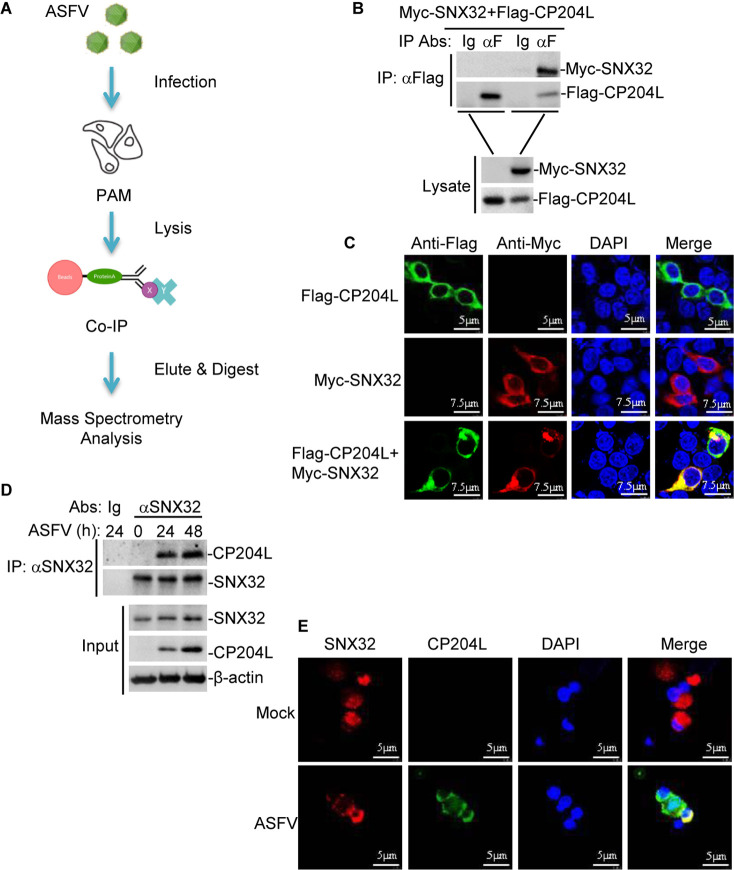
SNX32 interacts with ASFV CP204L. (**A**) A schematic representation of the IP-MS approach for identifying ASFV CP204L-host protein interactions in PAMs. (**B**) Interaction between ASFV Flag-CP204L and Myc-SNX32. Human embryonic kidney 293T (HEK-293T) cells were cotransfected with Flag-CP204L and Myc-SNX32 or empty vector for 24 h before coimmunoprecipitation and immunoblotting analysis with the indicated antibodies. (**C**) Colocalization of ASFV Flag-CP204L with Myc-SNX32. HEK-293T cells were cotransfected with Flag-CP204L and Myc-SNX32. Twenty-four hours after transfection, the cells were fixed and subjected to indirect immunofluorescence to detect Flag-CP204L (green) and Myc-SNX32 (red) with mouse anti-Myc and rabbit anti-Flag antibodies. The position of the nucleus is indicated by 4′,6-diamidino-2-phenylindole (DAPI; blue) staining in the merged image. (**D**) Endogenous SNX32 is associated with ASFV CP204L in PAMs. PAMs were infected with ASFV for the indicated times. Coimmunoprecipitation and immunoblotting analysis were performed with the indicated antibodies. (**E**) Endogenous colocalization of ASFV CP204L with SNX32. PAMs were infected or uninfected with ASFV for the indicated times. The cells were fixed and subjected to indirect immunofluorescence to detect CP204L (green) and SNX32 (red) with the indicated antibodies.

Since CP204L is an important immunogenic protein of ASFV, identifying the CP204L-host interaction can be valuable for understanding the mechanism of ASFV-host interaction. We previously performed immunoprecipitation and mass spectrometry (IP-MS) experiments to identify host proteins that interact with CP204L (Fig. 1A). Briefly, the cell lysates from ASFV-infected porcine alveolar macrophages (PAMs) were immunoprecipitated with mouse anti-CP204L monoclonal antibody or mouse IgG. Subsequently, the samples were subjected to liquid chromatography with tandem mass spectrometry (LC-MS/MS). Based on the protein discriminant scores, the top 10 hits, including SNX32, PSMC5, VIPR1, CAP1, and RAB1B, were listed in Table 1. In this study, we focused on SNX32 for further research due to its highest protein score，and the corresponding construction primers were all listed in Table 2.

**TABLE 1:** The information of the proteins identified by IP-MS

Rank	Protein name	Protein name description	Species	Accession no.	Mol. weight (kDa)	Sequence length	Protein score
1	SNX32	Sorting nexin 32	*Sus scrofa*	XM_003122527.4	44.895	393	38.368
2	PSMC5	Proteasome 26S subunit, ATPase 5	*Sus scrofa*	NM_213983.1	45.285	402	36.119
3	VIPR1	Vasoactive intestinal polypeptide receptor 1	*Sus scrofa*	NM_214036.1	44.335	392	34.983
4	CAP1	Adenylyl cyclase-associated protein	*Sus scrofa*	XM_021096009.1	51.409	475	34.811
5	RAB1B	Ras-related protein Rab-1B	*Sus scrofa*	DQ917628.1	20.542	184	34.513
6	SLC22A5	Solute carrier family 22 member 5	*Sus scrofa*	XM_021085433.1	147.19	1,256	34.32
7	ZNF483	Zinc finger protein 483	*Sus scrofa*	XM_021085145.1	84.395	737	34.262
8	RPS7	40S ribosomal protein S7	*Sus scrofa*	XM_005662783.3	20.762	183	34.062
9	FAM129C	Family with sequence similarity 129 member C	*Sus scrofa*	XM_005661181.3	45.643	406	33.658
10	ILK	Integrin-linked kinase	*Sus scrofa*	XM_003357131.4	45.679	401	33.644

**TABLE 2:** Primers used in this study^*a*^

Primer	Sequence	Description
HA-RAB1B-F	ACGCGTCGACCATGAACCCCGAATATGAC	For amplification of RAB1B
HA-RAB1B-R	ATAAGAATGCGGCCGCCTAGCAACAGCCGCCACCAG	
Myc-SNX32-F	AGACCCAAGCTGGCTAGTTAAGCTTATGGAGGAGCATCAAGAGGCTGG	For amplification of SNX32
Myc-SNX32-R	GTTTTTGTTCGAAGGGCCCTCTAGAAGGCTCCCCTTTGAGGATGACAA	
Flag-CP204L-F	GGAATTCATGGATTTTATTTTAAATATA	For amplification of CP204L
Flag-CP204L-R	GCTCTAGACTATTTTTTTTTTAAAAGTTTAATAACCATGAG	

^*a*^ F, forward; R, reverse.

We next investigated the interaction between SNX32 and CP204L. Transient transfection and coimmunoprecipitation experiments revealed that Myc-SNX32 interacted with Flag-CP204L (Fig. 1B). Furthermore, confocal microscopy showed that Myc-SNX32 colocalized with Flag-CP204L in the cytoplasm (Fig. 1C). In addition, we determined the interaction between SNX32 and CP204L upon ASFV infection. Coimmunoprecipitation experiments using the SNX32 antibody revealed that SNX32 was constitutively associated with CP204L in ASFV-infected PAMs (Fig. 1D). To confirm the colocalization of endogenous SNX32 with CP204L, PAMs were infected with ASFV and analyzed using confocal microscopy. Confocal images of the cells immunostained with anti-SNX32 and anti-CP204L antibodies showed colocalization between SNX32 and CP204L (Fig. 1E). Taken together, these results indicate that SNX32 interacts with CP204L.

### SNX32 inhibits ASFV replication

**Fig 2:**
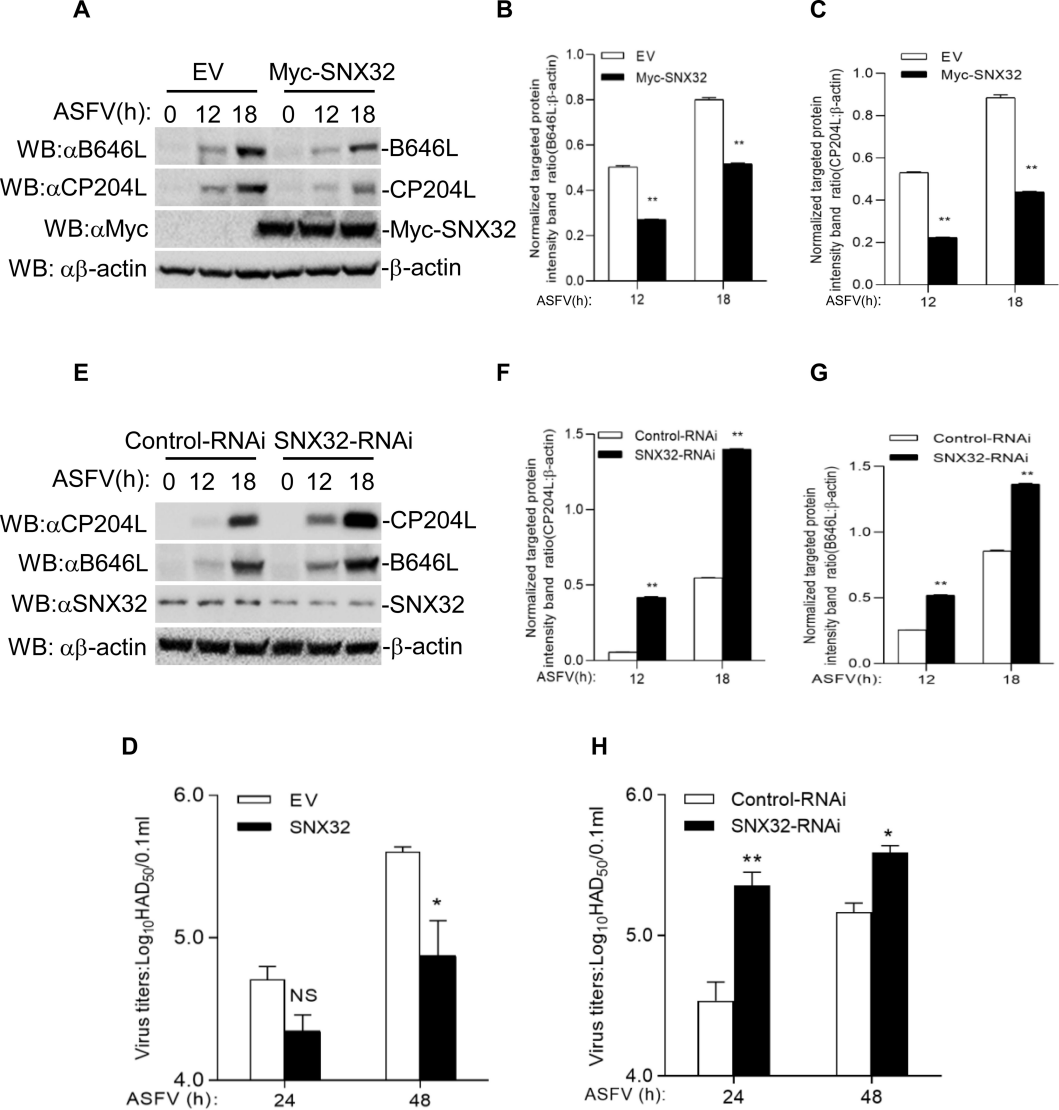
SNX32 inhibits ASFV replication. (**A**) Effect of SNX32 overexpression in MA104 cells on ASFV replication. SNX32 overexpression in MA104 cells was infected with ASFV at a multiplicity of infection (MOI) of 5.0 for the indicated times. Myc-SNX32, CP204L, and B646L proteins were detected by immunoblotting analysis with indicated antibodies. (**B and C**) The ratio of B646L or CP204L to β-actin was normalized to control conditions in (**A**). (**D**) Effect of SNX32 overexpression on ASFV titers in MA104 cells. SNX32 overexpression in MA104 cells was infected with ASFV (MOI = 5.0) for the indicated times. The virus titers in the supernatants collected were determined by the “rosettes” assay and expressed as hemadsorption (HAD)_50_/0.1 mL. The experiment shown is a representative experiment of three independent experiments with the mean ± SD of three technical replicates. (**E**) Effect of SNX32-RNAi on ASFV proteins in PAMs. SNX32 knockdown in PAMs was infected with ASFV (MOI = 5.0) for the indicated times. SNX32, CP204L, and B646L proteins were detected by immunoblotting analysis with the indicated antibodies. (**F and G**) The ratio of B646L or CP204L to β-actin was normalized to control conditions in (**E**). (**H**) Effect of SNX32-RNAi on ASFV titers in PAMs. SNX32 knockdown in PAMs was infected with ASFV (MOI = 5.0) for the indicated times. The virus titers in the supernatants collected were determined by the “rosettes” assay and expressed as HAD_50_/0.1 mL. The experiment shown is a representative experiment of three independent experiments with the mean ± SD of three technical replicates.

It has been reported that African green monkey cells MA104 is a suitable substrate for virus isolation of ASFV, and ASFV could be isolated from infected blood samples (29, 30). To further evaluate the importance of SNX32 in host defense against ASFV, MA104 cells were transfected with equal amounts of Myc-SNX32 or empty vector plasmids for 24 h, and then, the cells were infected with ASFV for another 12 or 18 h. The expression of viral protein was then analyzed by western blotting. The result showed that the overexpression of Myc-SNX32 obviously suppressed the protein abundance of ASFV B646L and CP204L compared with cells transfected with the empty vector plasmid during ASFV infection (Fig. 2A to C). Next, the virus in the culture supernatant was measured to detect the virus yields. As expected, virus yields significantly decreased after 48 h post-infection in the Myc-SNX32-overexpressing cells compared with empty vector-transfected cells (Fig. 2D).

Furthermore, we evaluated ASFV replication in SNX32 knockdown PAMs through small interfering RNA (siRNA). PAMs were transfected with the negative control siRNA (NC siRNA) or SNX32 siRNA for 36 h and then infected with equal amounts of ASFV for another 12 or 18 h. The viral protein and viral yields were measured. Knockdown of SNX32 resulted in a visible increase in viral protein expression (Fig. 2E through G) and virus yields (Fig. 2H). These results suggest that SNX32 negatively regulates ASFV replication and growth.

### SNX32 mediates the autophagy degradation of CP204L

**Fig 3:**
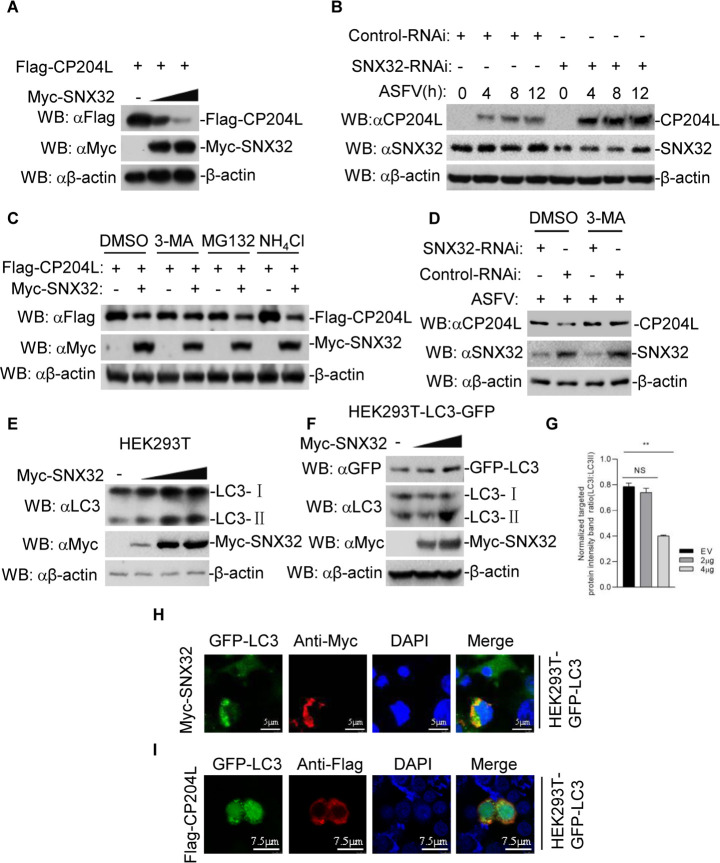
SNX32 degrades CP204L through the autophagy pathway. (**A**) Dose-dependent effect of SNX32 on the expression of CP204L in HEK-293T cells. HEK-293T cells (2 × 10^5^) were transfected with the Flag-CP204L (0.5 µg) and Myc-SNX32 plasmids (0, 0.5, or 1 µg) for 24 h. The cell lysates were analyzed by immunoblotting with anti-β-actin, anti-Flag, and anti-Myc antibodies. (**B**) Effect of SNX32 knockdown on the expression of CP204L in PAMs. PAMs were transfected with NC siRNA or SNX32 siRNA for 36 h and then left uninfected or infected with ASFV for the indicated times. Then, the samples were analyzed by immunoblotting with the indicated Abs. (**C**) SNX32 mediates the autophagy pathway degradation of CP204L in HEK-293T cells. HEK-293T cells (2 × 10^5^) were transfected with the indicated plasmids. Eighteen hours after transfection, the cells were treated with the indicated inhibitors [MG132 (25 µM), 3-MA (0.5 µg/µL), and NH_4_Cl (25 mM)] for 6 h before immunoblotting analysis. (**D**) Effect of autophagy inhibitor 3-MA on SNX32-mediated destabilization of CP204L. PAMs were transfected with NC siRNA or SNX32 siRNA for 36 h and then treated with 3-MA (0.5 µg/µL) for 6 h. And then, the cells were left uninfected or infected with ASFV for 12 h and then analyzed by immunoblotting with the indicated Abs. (**E**) Dose-dependent effect of SNX32 on the endogenous expression of LC3 in HEK-293T cells. HEK-293T cells (2 × 10^5^) were transfected with the Myc-SNX32 (0, 0.5, 1, or 2 µg) for 24 h. The cell lysates were analyzed by immunoblotting with anti-β-actin, anti-LC3, and anti-Myc antibodies. (**F**) Dose-dependent effect of SNX32 on the endogenous expression of LC3 in HEK-293T-LC3-GFP cells. HEK-293T-LC3-GFP cells (2 × 10^5^) were transfected with the Myc-SNX32 (0, 2, or 4 µg) for 24 h. The cell lysates were analyzed by immunoblotting with anti-GFP, anti-LC3, anti-Myc, and anti-β-actin antibodies. (**G**) The ratio of LC3-I to LC3-II was normalized to control conditions in (**F**). (**H and I**) Colocalization of Myc-SNX32 or Flag-CP204L protein with GFP-LC3. HEK-293T-GFP-LC3 cells were transfected with Myc-SNX32 (**H**) or Flag-CP204L (**I**). One day after transfection, the cells were fixed and subjected to indirect immunofluorescence to detect GFP-LC3 (green) and Myc-SNX32 (red, **H**) or Flag-CP204L (red, **I**) with mouse anti-Myc or anti-Flag antibody. The position of the nucleus is indicated by DAPI (blue) staining in the merged image.

We next investigated the effect of SNX32 on the expression of CP204L. In transient transfection experiments, Myc-SNX32 inhibited the expression of Flag-CP204L in a dose-dependent manner in HEK-293T cells (Fig. 3A). Additionally, we examined the effect of SNX32 on the expression of CP204L during ASFV infection. PAMs were transfected with NC siRNA or SNX32 siRNA for 36 h, and then infected with ASFV for the indicated times. The result showed that SNX32 knockdown stabilized the expression of CP204L (Fig. 3B).

The ubiquitin-proteasome and autophagy-lysosome pathways are two different systems that regulate protein degradation in eukaryotic cells (31). To investigate the mechanisms responsible for the role of SNX32 on the stability of CP204L, HEK-293T cells were transfected with the indicated plasmids and then treated with various inhibitors for protein degradation pathways. The result showed that Myc-SNX32-mediated degradation of Flag-CP204L was completely restored by the autophagy inhibitor 3-methyladenine (3-MA), but not by the lysosomal inhibitor NH_4_Cl or the proteasome inhibitor MG132 (Fig. 3C). We further investigated whether 3-MA could restore the expression of CP204L in the context of ASFV infection. The result indicated that 3-MA restored the expression of CP204L during ASFV infection (Fig. 3D). Collectively, these results suggest that SNX32 mediated the degradation of CP204L by the autophagy pathway.

LC3 has been widely used as a marker to track the formation of autophagosomes and monitor autophagy (32). To further investigate the effect of SNX32 on autophagy, we examined the conversion of endogenous LC3-I to LC3-II. In transient transfection experiments, Myc-SNX32 overexpression increased LC3-II conversion (Fig. 3E). Furthermore, the HEK-293T-GFP-LC3 cell lines stably expressing GFP-LC3 were constructed to evaluate the effect of SNX32 on autophagy. Similarly, the overexpression of Myc-SNX32 increased the conversion of LC3-I to LC3-II compared with the control (Fig. 3F and G). Moreover, the ring-like and small puncta GFP-LC3 structures were also observed in Myc-SNX32 or Flag-CP204L-overexpressed HEK-293T-GFP-LC3 cells (Fig. 3H and I).

### SNX32 interacts with the autophagy-associated protein RAB1B

**Fig 4:**
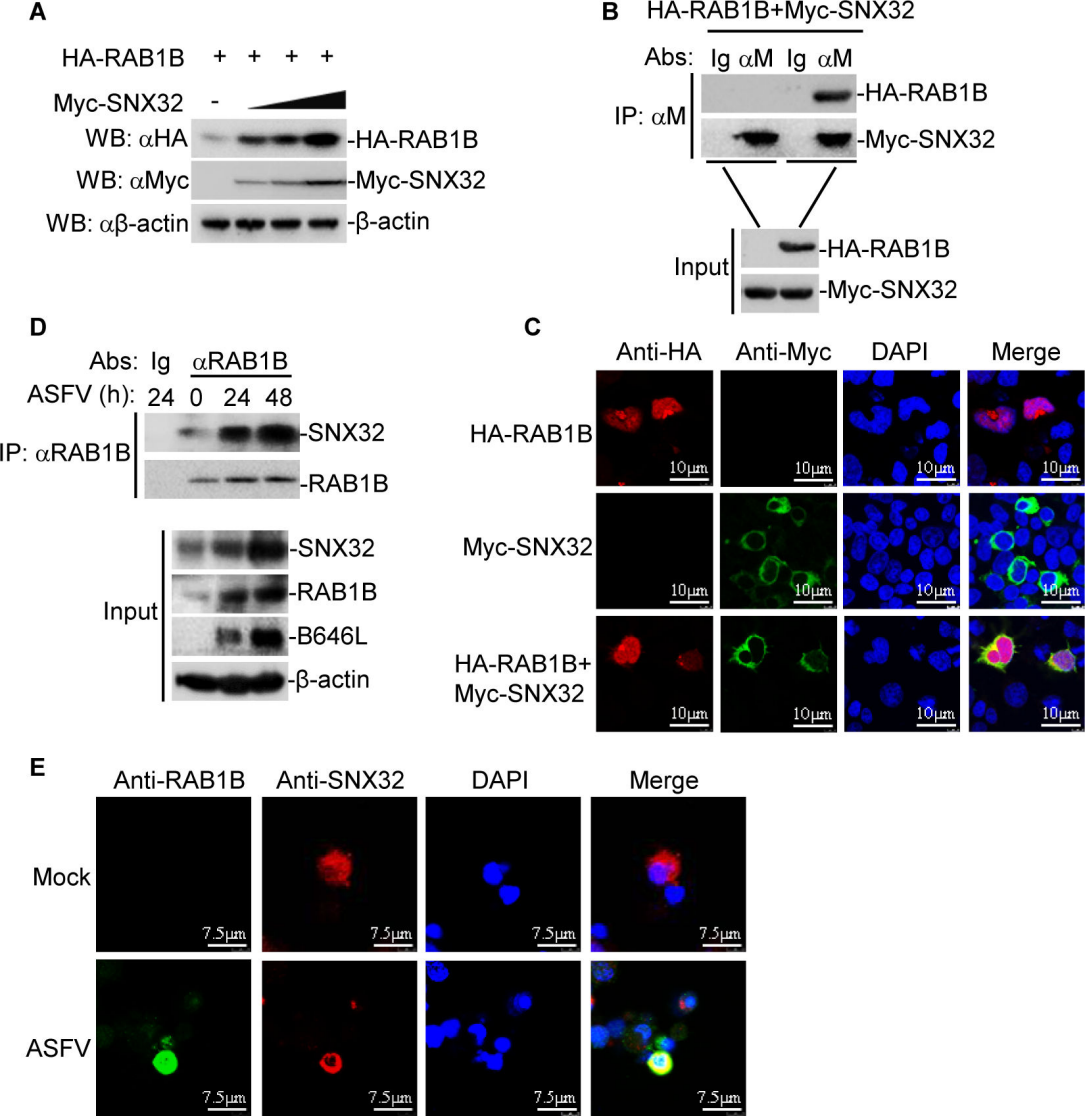
SNX32 interacts with RAB1B and promotes the expression of RAB1B. (**A**) Dose-dependent effect of SNX32 on the expression of RAB1B in HEK-293T cells. HEK-293T cells (2 × 10^5^) were transfected with the Myc-SNX32 (0, 0.5, 1, or 2 µg) and HA-RAB1B plasmids (0.5 µg) for 24 h. The cell lysates were collected and analyzed by immunoblotting with anti-HA, anti-Myc, or anti-β-actin antibodies. (**B**) The interaction of Myc-SNX32 and HA-RAB1B. HEK-293T cells were cotransfected with HA-RAB1B and Myc-SNX32 for 24 h and then subjected to coimmunoprecipitation and immunoblotting analysis with the indicated antibodies. (**C**) Colocalization of HA-RAB1B with Myc-SNX32. HEK-293T cells were cotransfected with HA-RAB1B and Myc-SNX32. Twenty-four hours after transfection, the cells were fixed and subjected to indirect immunofluorescence to detect Myc-SNX32 (green) and HA-RAB1B (red) with mouse anti-Myc and rabbit anti-HA antibodies. The position of the nucleus is indicated by DAPI (blue) staining in the merged image. (**D**) Endogenous SNX32 is associated with RAB1B in PAMs. PAMs were infected with ASFV for the indicated times. Coimmunoprecipitation and immunoblotting analysis were performed with the indicated antibodies. (**E**) Endogenous colocalization of RAB1B with SNX32. PAMs were infected with ASFV for the indicated times. The cells were fixed and subjected to indirect immunofluorescence to detect RAB1B (green) and SNX32 (red) with the indicated antibodies.

Next, we examined whether autophagy-associated proteins participated in SNX32-mediated degradation. Our previous IP-MS results identified cellular RAB1B as a potential target of ASFV CP204L. In transient transfection experiments, we observed that Myc-SNX32 promoted HA-RAB1B expression in a dose-dependent manner in HEK-293T cells (Fig. 4A). Coimmunoprecipitation experiments showed that Myc-SNX32 interacted with HA-RAB1B in HEK-293T cells (Fig. 4B). To examine the colocalization of RAB1B with SNX32, HEK-293T cells were cotransfected with plasmids expressing HA-RAB1B and Myc-SNX32, and the subcellular localization of RAB1B with SNX32 was examined by confocal microscopy. The results indicated that HA-RAB1B colocalized with Myc-SNX32 (Fig. 4C). These results lead to the hypothesis that RAB1B may deliver CP204L to the autophagosomes for degradation. Furthermore, endogenous coimmunoprecipitation experiments indicated that RAB1B was associated with SNX32 in ASFV-infected PAMs (Fig. 4D). To confirm that endogenous RAB1B colocalized with SNX32, PAMs were infected with ASFV and analyzed by confocal microscopy. Confocal images of the cells immunostained with anti-RAB1B and anti-SNX32 antibodies showed colocalization of RAB1B with SNX32 (Fig. 4E). Collectively, these findings demonstrate that SNX32 interacts with RAB1B and enhances the expression of RAB1B.

### RAB1B mediates the degradation of CP204L

**Fig 5:**
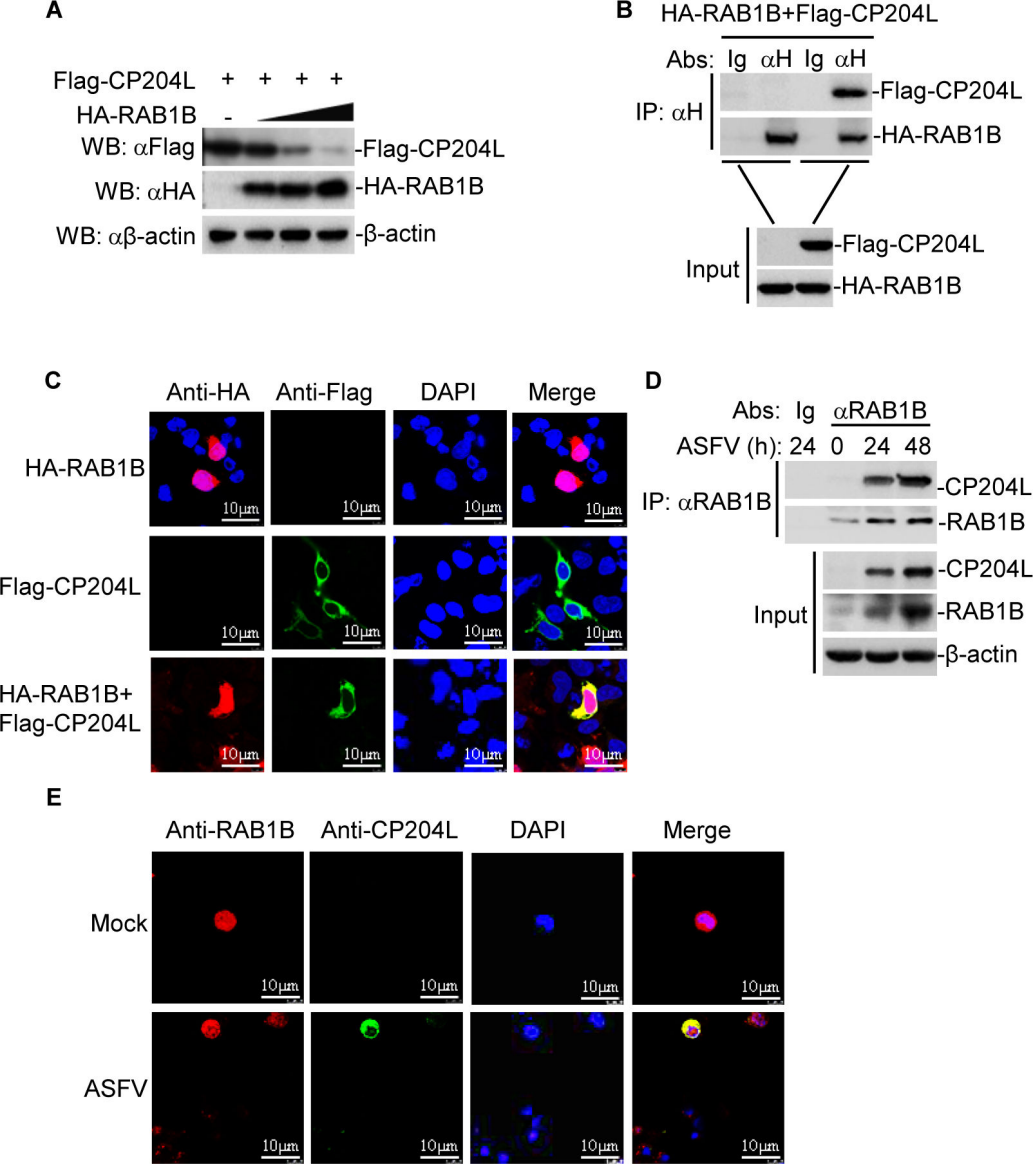
RAB1B degrades CP204L and interacts with CP204L. (**A**) Dose-dependent effect of RAB1B on the expression of ASFV CP204L in HEK-293T cells. HEK-293T cells (2 × 10^5^) were transfected with the Flag-CP204L (0.5 µg) and HA-RAB1B plasmids (0, 0.5, 1, or 2 µg) for 24 h. The cell lysates were analyzed by immunoblotting with anti-β-actin and anti-Flag or anti-HA antibodies. (**B**) HEK-293T cells were transfected with Flag-CP204L and HA-RAB1B for 24 h before coimmunoprecipitation and immunoblotting analysis with the indicated antibodies. (**C**) Colocalization of ASFV Flag-CP204L with HA-RAB1B. HEK-293T cells were cotransfected with Flag-CP204L and HA-RAB1B. Twenty-four hours after transfection, the cells were fixed and subjected to indirect immunofluorescence to detect Flag-CP204L (green) and HA-RAB1B (red) with mouse anti-Flag and rabbit anti-HA antibodies. The position of the nucleus is indicated by DAPI (blue) staining in the merged image. (**D**) Endogenous RAB1B is associated with ASFV CP204L in PAMs. PAMs were infected with ASFV for the indicated times. Coimmunoprecipitation and immunoblotting analysis were performed with the indicated antibodies. (**E**) Endogenous colocalization of ASFV CP204L with RAB1B. PAMs were infected with ASFV for the indicated times. The cells were fixed and subjected to indirect immunofluorescence to detect CP204L (green) and RAB1B (red) with the indicated antibodies.

Subsequently, we determined the effect of RAB1B on the expression of CP204L. In the transient transfection experiments, we observed that HA-RAB1B mediated the degradation of Flag-CP204L in a dose-dependent manner in HEK-293T cells (Fig. 5A). Coimmunoprecipitation experiments showed that HA-RAB1B interacted with Flag-CP204L in HEK-293T cells (Fig. 5B). To examine the colocalization of RAB1B with CP204L, HEK-293T cells were cotransfected with plasmids expressing HA-RAB1B and Flag-CP204L, and the subcellular localization of RAB1B with CP204L was examined by confocal microscopy. The result indicated that HA-RAB1B colocalized with Flag-CP204L (Fig. 5C). Furthermore, endogenous coimmunoprecipitation experiments indicated that RAB1B was associated with CP204L in ASFV-infected PAMs (Fig. 5D). To confirm that endogenous RAB1B colocalized with CP204L, PAMs were infected with ASFV and analyzed by confocal microscopy. Confocal images of the cells immunostained with anti-RAB1B and anti-CP204L antibodies showed colocalization of RAB1B with CP204L (Fig. 5E). Collectively, these findings demonstrate that RAB1B interacts with CP204L and impairs the expression of CP204L.

### RAB1B inhibits ASFV replication

**Fig 6:**
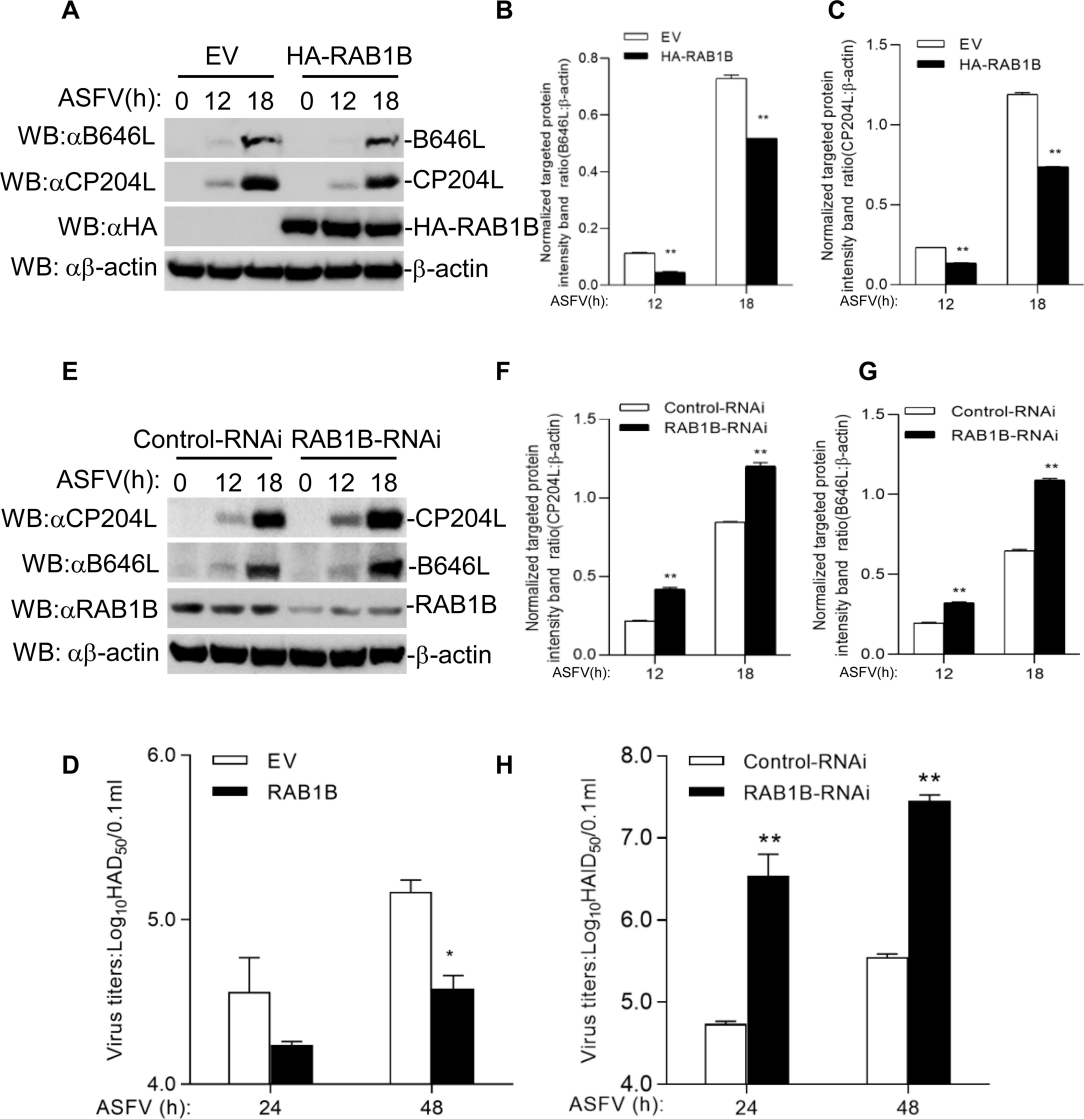
RAB1B inhibits ASFV replication. (**A**) Effect of RAB1B overexpression in MA104 cells on ASFV replication. RAB1B overexpression in MA104 cells was infected with ASFV (MOI = 5.0) for the indicated times. HA-RAB1B, CP204L, and B646L proteins were detected by immunoblotting analysis with the indicated antibodies. The ratio of B646L or CP204L to β-actin was normalized. (**B and C**) The ratio of B646L or CP204L to β-actin was normalized to control conditions in (**A**). (**D**) Effect of RAB1B overexpression on ASFV titers in MA104 cells. RAB1B overexpression in MA104 cells was infected with ASFV (MOI = 5.0) for the indicated times. The virus titers in the supernatants collected were determined by the “rosettes” assay and expressed as HAD_50_/0.1 mL. The experiment shown is a representative experiment of three independent experiments with the mean ± SD of three technical replicates. (**E**) Effect of RAB1B-RNAi on ASFV proteins in PAMs. RAB1B knockdown in PAMs was infected with ASFV (MOI = 5.0) for the indicated times. RAB1B, CP204L, and B646L proteins were detected by immunoblotting analysis with the indicated antibodies. The ratio of B646L or CP204L to β-actin was normalized. (**F and G**) The ratio of B646L or CP204L to β-actin was normalized to control conditions in (**E**). (**H**) Effect of RAB1B-RNAi on ASFV titers in PAMs. RAB1B knockdown in PAMs was infected with ASFV (MOI = 5.0) for the indicated times. The virus titers in the supernatants collected were determined by the “rosettes” assay and expressed as HAD_50_/0.1 mL. The experiment shown is a representative experiment of three independent experiments with the mean ± SD of three technical replicates.

To further evaluate the importance of RAB1B during ASFV infection, we transfected MA104 cells with empty vector and HA-RAB1B plasmids and then infected with equal amounts of ASFV for 12 and 18 h at 24 h post-transfection. The expression of viral protein was detected by western blotting. A visual reduction in viral protein B646L and CP204L were observed in the RAB1B-overexpressing cells compared with those transfected with empty vector plasmids (Fig. 6A through C). The virus in the cell culture supernatant was also measured. As expected, the virus yields observably decreased in the RAB1B-overexpressing cells compared with the empty vector-transfected cells (Fig. 6D).

Furthermore, the viral protein and virus yields in the RAB1B knockdown cells were measured. The knockdown of RAB1B was performed by transfection with siRNA that targets RAB1B. PAMs were transfected with NC siRNA or RAB1B siRNA for 36 h and then infected with equal amounts of ASFV for another 12 and 18 h. Knockdown of RAB1B increased the viral protein expression (Fig. 6E through G) and virus yields in ASFV-infected PAMs (Fig. 6H). These results indicate that RAB1B plays an antiviral role during ASFV infection.

### SNX32 promotes RAB1B-mediated autophagy degradation of CP204L

**Fig 7:**
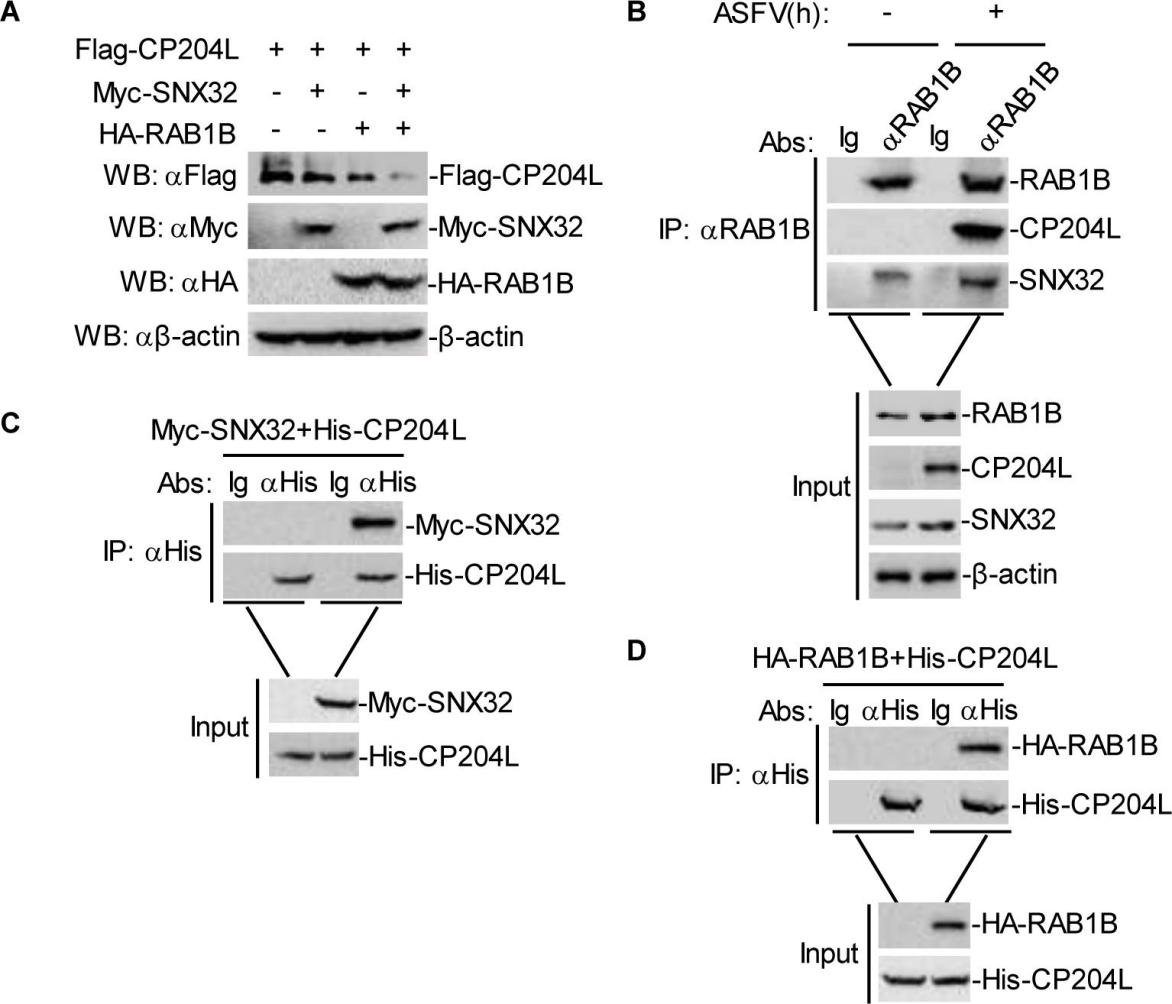
RAB1B potentiates SNX32-mediated degradation of CP204L. (**A**) Effect of RAB1B and SNX32 on the expression of ASFV CP204L in HEK-293T cells. HEK-293T cells (2 × 10^5^) were transfected with the Flag-CP204L (0.5 µg) and HA-RAB1B plasmids (2 µg) and Myc-SNX32 (2 µg) for 24 h. The cell lysates were analyzed by immunoblotting with anti-β-actin, anti-Flag, and anti-Myc or anti-HA antibodies. (**B**) Endogenous RAB1B is associated with SNX32 and ASFV CP204L in PAMs. PAMs were infected with ASFV (MOI = 5.0) for the indicated times. Coimmunoprecipitation and immunoblotting analysis were performed with the indicated antibodies. (**C**) Ni-NTA pull-down analysis of the interaction of SNX32 with CP204L. HEK-293T cells (2 × 10^5^) were transfected with the Myc-SNX32 (5 µg) for 24 h, and Ni-NTA pull-down and immunoblotting analysis were performed with the indicated antibodies. (**D**) Ni-NTA pull-down analysis of the interaction of RAB1B with CP204L. HEK-293T cells (2 × 10^5^) were transfected with the HA-RAB1B (5 µg) for 24 h, and Ni-NTA pull-down and immunoblotting analysis were performed with the indicated antibodies.

Next, we examined whether and how RAB1B mediated the autophagic degradation of CP204L. Cotransfection experiments showed that Myc-SNX32 potentiated HA-RAB1B-mediated autophagic degradation of Flag-CP204L (Fig. 7A). In endogenous coimmunoprecipitation experiments, SNX32 and RAB1B were found to be associated with CP204L in PAMs following ASFV infection (Fig. 7B). To further demonstrate the interaction relationship between SNX32, CP204L, and RAB1B, we examined if CP204L physically interacts with SNX32 and RAB1B; immunoprecipitation and Ni-NTA pull-down experiments were performed. The results indicated that Myc-SNX32 and HA-RAB1B could be pulled down with His-tagged-CP204L (Fig. 7C and D), indicating a physical interaction of CP204L with SNX32 and RAB1B.

**Fig 8:**
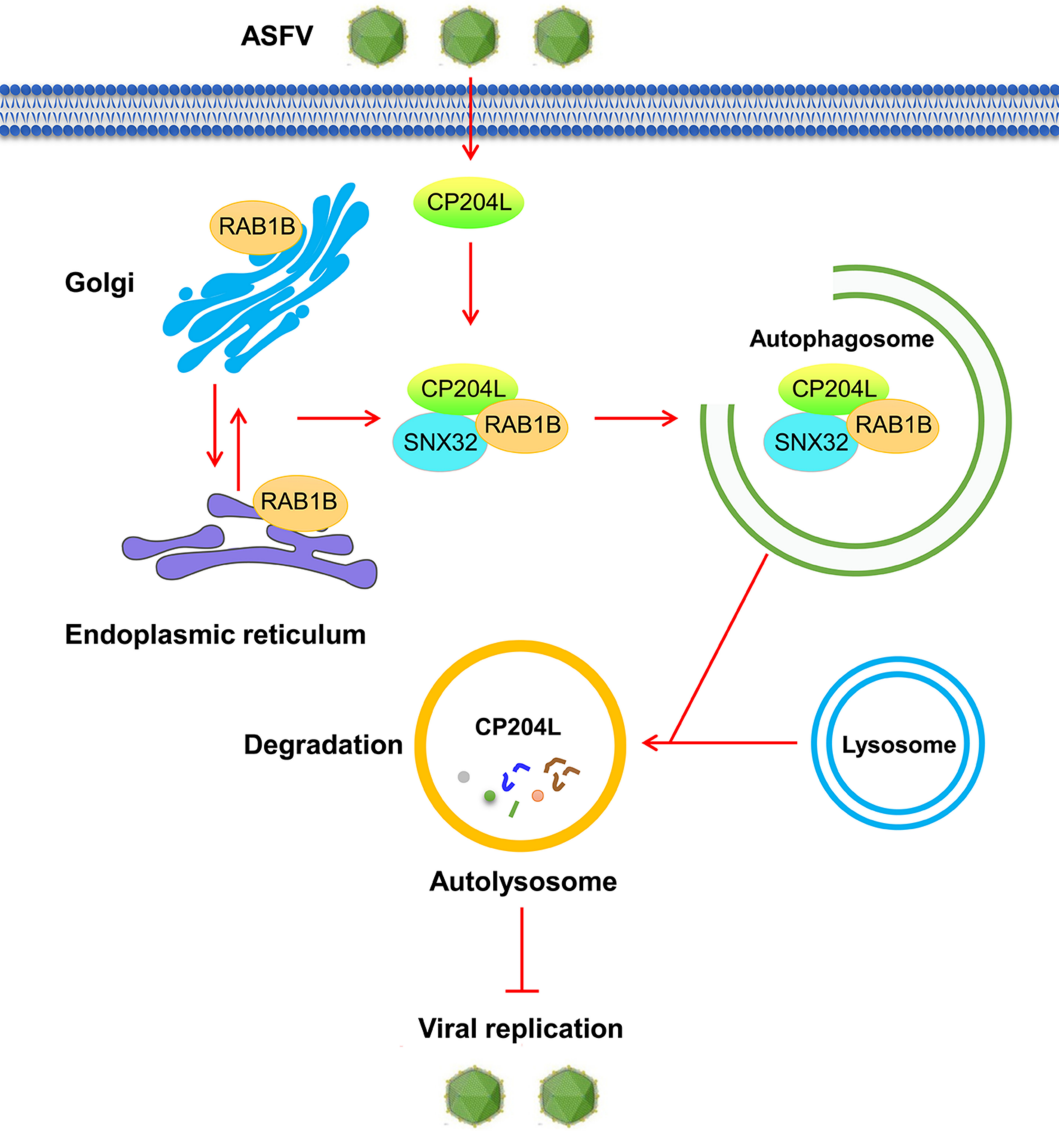
A working model for the regulation of the stability of ASFV CP204L by host protein SNX32. During ASFV infection, SNX32, ASFV CP204L, and RAB1B form a complex. Subsequently, SNX32 interacts with autophagy-related protein RAB1B and promotes the expression of RAB1B; RAB1B interacts with CP204L and degrades CP204L, which ultimately leads to the reduction of CP204L and inhibition of the replication of ASFV.

Based on these findings, we propose a working model of SNX32-mediated degradation of ASFV CP204L (Fig. 8). During ASFV infection, SNX32, ASFV CP204L, and RAB1B form a complex. Subsequently, SNX32 interacts with autophagy-related protein RAB1B and promotes the expression of RAB1B; RAB1B interacts with CP204L and degrades CP204L, which ultimately leads to the reduction of CP204L and inhibits the replication of ASFV.

## DISCUSSION

Autophagy, a lysosomal degradation pathway, plays an essential role in multiple aspects of immunity, including immune system development, regulation of innate and adaptive immune and inflammatory responses, selective degradation of intracellular microbes, and host protection against infectious diseases (33–36). Until now, it has been reported that many ASFV proteins are involved in regulating autophagy. On the one hand, some ASFV proteins activate autophagy by interacting with the innate immune signaling molecules. For instance, I215L interacts with IRF9 and induces IRF9 degradation through the autophagy-lysosome pathway, thereby inhibiting type I IFN signaling and counteracting the host innate immune response (37); A137R inhibits type I interferon production via the autophagy-mediated lysosomal degradation of TBK1 (38); MGF505-7R interacts with IRF7, degrading IRF7 through the autophagy, cysteine, and proteasome pathways (39); and MGF505-7R promotes the expression of the autophagy-related protein ULK1 to degrade STING, thus inhibiting the cGAS-STING signaling pathway (40). On the other hand, ASFV proteins activate autophagy by interacting with host proteins. For example, E199L downregulates the expression of PYCR2, resulting in autophagy activation (41); A179L inhibits autophagy by binding to Beclin1 and prevents autophagosome formation during nutrient deprivation (42); and K205R inhibits serine/threonine kinase 1 and the mechanistic target of the rapamycin kinase signaling pathway, thereby activating unc-51-like autophagy-activating kinase 1 and, hence, autophagy (43). However, there are few reports on how host proteins regulate ASFV replication through autophagy.

Rab GTPases, which belong to the Ras superfamily of small GTPases, have emerged as central regulators of vesicle budding, motility, and fusion (44). The Rab protein is a small GTPase that belongs to the Ras-like GTPase superfamily and regulates the vesicle traffic process. Numerous Rab proteins have been shown to be involved in various stages of autophagy (45). Among them, Rab1, Rab5, Rab7, Rab9A, Rab11, Rab23, Rab32, and Rab33B participate in autophagosome formation, whereas Rab9 is required in noncanonical autophagy. Rab7, Rab8B, and Rab24 have a key role in autophagosome maturation (45). More recently, Rab1 has emerged as a common target required for the intracellular survival of many pathogens (46, 47). Rab1 has two isoforms, Rab1a and Rab1b, which share 92% amino acid similarity and are thought to be functionally redundant (48–50). Both isoforms have been shown to be involved in ER-to-Golgi trafficking (51). During the infection of macrophages by *Yersinia pestis,* Rab1b recruitment to the *Yersinia*-containing vacuole directly inhibits phagosome maturation (48); active Rab1b stabilizes Arf1 on Golgi membranes, and Rab1b is required for GBF1 membrane association (50). Rab1B is associated with ATG9A vesicles and regulates autophagosome formation (52). Overall, these studies suggest that RAB1B is important in the transport of membranes from the ER to the Golgi. In this study, we found that SNX32 degraded CP204L through the autophagy-mediated pathway. Meanwhile, we screened RAB1B from the IP-MS results of CP204L. In addition, we confirmed the interaction between SNX32 and RAB1B and demonstrated the association of CP204L and RAB1B. The coimmunoprecipitation results indicated an interaction among SNX32, RAB1B, and CP204L.

There is little research on the interaction between host proteins and CP204L. ASFV CP204L interacts with VPS39, blocking its association with the lysosomal HOPS complex, and loss of VPS39 reduces the levels of virus proteins synthesized in the early phase of infection and delays ASFV replication (17). The interaction of CP204L with RPSA, DAB2, CAPG, and ARPC5 might be involved in the viral internalization process mediated by clathrin and micropinocytosis, and the interaction of CP204L with DAB2, PARP9, RPSA, OAS1, and VBP1 may be related to innate immune response. However, the specific mechanism remains unclear (18). Further research is required to investigate the function of CP204L.

In conclusion, our study revealed that the mechanism of host protein SNX32 inhibited the replication of ASFV, and SNX32 mediated CP204L degradation through the RAB1B-dependent autophagy pathway. On the one hand, the results demonstrated that host protein could inhibit ASFV replication by degrading viral proteins. Thus, we could knock out some key host factors in ASFV-susceptible cell lines, thereby increasing the titer of the virus. In general, this study identified a potential target for the development of novel antiviral therapeutic strategies. On the other hand, based on current research findings, many viral proteins participated in the regulation of autophagy, such as A137R, MGF-505-7R, K205R, L83L, E199L, A179L, and MGF-110-9L (38, 40–43, 53, 54). Thus, we concluded that autophagy may play an important role in the regulation of ASFV replication, and the mechanism between viral protein and autophagy deserves further study.

## MATERIALS AND METHODS

### Cell culture, transfection, and virus infection

PAMs were prepared by bronchoalveolar lavage as previously described (55) and grown in RPMI 1640 medium supplemented with 2 mM L-glutamine, 100 U/mL gentamicin, nonessential amino acids, and 10% fetal bovine serum (FBS) [Biological Industries (BI)] at a humidified 37°C and 5% CO_2_ atmosphere. HEK-293T and MA104 cells were obtained from the American Type Culture Collection and grown in Dulbecco's modified Eagle medium (DMEM) supplemented with 2 mM L-glutamine, 100 U/mL gentamicin, nonessential amino acids, and 10% FBS. The ASFV isolates CN/GS/2018 were propagated on PAMs as previously described (56). ASFV CN/GS/2018 (wt-ASFV) was provided by the Lanzhou Veterinary Research Institute, Chinese Academy of Agricultural Sciences.

### Constructs

Mammalian expression plasmids for HA-tagged RAB1B and Myc-tagged SNX32 were amplified from the cDNA of PAMs and then constructed by standard molecular biology techniques. LC3-GFP was amplified from the cDNA of HEK-293T cells and then constructed by standard molecular biology techniques. To construct Flag-CP204L, a DNA fragment was amplified by PCR from the cDNA of ASFV-WT (ASFV CN/GS/2018), which contains full-length CP204L, and subcloned into the pCMV-3×Flag vector. The primers for amplification of plasmids are listed in Table 2.

### Antibodies and reagents

The mouse polyclonal antibody against CP204L and rabbit polyclonal antibody against B646L were prepared in our laboratory. Polyclonal rabbit anti-SNX32 (catalog no. 25763-1-AP), rabbit anti-p62 (catalog no. 18420-1-AP), and rabbit anti-RAB1B (catalog no. 17824-1-AP) were purchased from Proteintech. Rabbit anti-LC3B (catalog no. 3868s) and mouse anti-Myc (catalog no. 2276S) were purchased from Cell Signaling Technology. Monoclonal mouse anti-HA (catalog no. H3663), mouse IgG polyclonal antibody (catalog no. 12-371), rabbit IgG polyclonal antibody (catalog no. 12-370), and mouse anti-Flag (catalog no. F1804) were purchased from Sigma-Aldrich. Mouse anti-β-actin was purchased from Santa Cruz (catalog no. sc-58673). Alexa Fluor 488-conjugated goat anti-mouse IgG (H+L) (catalog no. 4408s) and Alexa Fluor 594-conjugated goat anti-rabbit IgG (H+L) (catalog no. 8889s) antibodies were purchased from Cell Signaling Technology; IPKine goat anti-mouse IgG heavy chain secondary antibody, HRP labeling (elimination of light chain interference) (catalog no. A25112) and IPKine goat anti-mouse IgG light chain secondary antibody, HRP labeling (elimination of heavy chain interference) (catalog no. A25012) were purchased from Abbkine. Dimethyl sulfoxide, 3-MA (autophagosome formation inhibitor), MG132 (proteasome inhibitor), and NH_4_Cl (lysosome inhibitor) were purchased from Sigma-Aldrich.

### Generation of LC3 overexpression cell lines

To generate LC3 overexpression cell lines, LC3 sequence was cloned into the pCDH-CMV-MCS-EF1-Puro vector and cotransfected into HEK-293T cells with packaging plasmids pLP1, pLP2, and pVSV-G (57) for 24 h using Lipofectamine 2000. The medium was refreshed 12 h after transfection. At 48 and 72 h post-transfection, cell supernatants containing lentivirus were collected and filtered with a 0.45-µm filter. HEK-293T cells were infected with lentivirus for 24 h, followed by selection with puromycin (1 µg/mL, Amresco) for 14 days. Thus, an LC3-GFP overexpression cell line was obtained.

### Virus titration

Wild-type ASFV CN/GS/2018 was quantified using the hemadsorption assay as described previously (58) with minor modifications. PAMs were seeded in 96-well plates. The samples were then added to the plates and titrated in triplicate using 10-fold serial dilutions. HAD was determined on day 7 post-inoculation, and 50% HAD doses (HAD_50_) were calculated using the method of Reed and Muench (40).

### RNA interference experiments

Small interfering RNAs corresponding to the porcine SNX32 and RAB1B target sequence were purchased from Sangon Biotech (Shanghai, China). PAMs (1 × 10^5^) were transfected with control siRNA, SNX32 siRNA, or RAB1B siRNA (2 µg) for 36 h; then, the cells were left uninfected or infected with ASFV for the indicated times. The expression of proteins was detected by immunoblot using the indicated Abs.

### Confocal microscopy

HEK-293T cells were transfected with the indicated Myc-SNX32 (1 µg) and Flag-CP204L (1 µg) plasmids using Lipofectamine 2000 (Invitrogen). At 24 h after transfection, the cells were fixed with 4% paraformaldehyde for 10 min at room temperature and permeabilized with 0.1% Triton X-100 for 15 min. The cells were incubated with anti-Myc mouse monoclonal or anti-Flag rabbit monoclonal antibodies for 2 h. The cells were then incubated with goat anti-mouse IgG (whole molecule)-FITC antibody and goat anti-rabbit IgG (whole molecule)-tetramethyl rhodamine isothiocyanate antibody. Cells were stained with DAPI for 15 min and examined with a Leica SP2 confocal system (Leica Microsystems).

### Coimmunoprecipitation and immunoblotting analysis

For the transient transfection coimmunoprecipitation experiments, HEK-293T cells were transfected with the appropriate plasmid. Twenty-four hours after transfection, the cells were harvested and lysed in 1 mL lysis buffer [20 mM Tris (pH 7.5), 150 mM NaCl, 1% Triton, 1 mM EDTA, 10 µg/mL aprotinin, 10 µg/mL leupeptin, and 1 mM PMSF (phenylmethanesulfonyl fluoride)]. For each immunoprecipitation reaction, 0.4 mL of cell lysate was incubated with 0.5 µg of the indicated antibody or control IgG and 40 µL protein G agarose beads (Santa Cruz Biotechnology) at 4°C. After 4 h incubation, the beads were washed three times with 1 mL lysis buffer containing 0.5 M NaCl. Samples were resolved by sodium dodecyl sulfate-polyacrylamide gel electrophoresis (SDS-PAGE) and transferred to Immobilon-P membranes (Millipore). The membranes were incubated with the indicated specific primary antibodies diluted in Tris buffered saline (TBS) supplemented with 1% milk. The membranes were washed three times with TBS and exposed for 1 h to specific horseradish peroxidase-conjugated secondary antibodies. Chemiluminescence detection was performed by ECL Prime (Millipore). For the endogenous coimmunoprecipitation experiments, PAMs were uninfected or infected with ASFV for the indicated times. The subsequent procedures were performed as described above.

### Immunoprecipitation and mass spectrometry

PAMs were infected with ASFV for 24 and 48 h, respectively; the cells were harvested and lysed in 1 mL lysis buffer [20 mM Tris (pH 7.5), 150 mM NaCl, 1% Triton, 1 mM EDTA, 10 µg/mL aprotinin, 10 µg/mL leupeptin, and 1 mM PMSF]. For each immunoprecipitation reaction, 0.4 mL cell lysate was incubated with 0.5 µg of the indicated antibody or control IgG and 40 µL protein G agarose beads (Santa Cruz Biotechnology) at 4℃. After 6–8 h incubation, the beads were washed six times with 1 mL lysis buffer containing 0.5 M NaCl. Then, the samples were added with 60 µL 2× loading buffer and heated at 100℃ for 10 min, followed by mass spectrometry analysis by Shanghai Hoogen Biological Company.

### Ni-NTA pull-down assays

Ni-NTA pull-down assays were performed as described (31, 59). Briefly, HEK-293T cells cultured in a 10-cm dish were transfected with the indicated plasmids. Twenty-four hours after transfection, the cells from each dish were collected and divided into two aliquots. One aliquot was lysed in lysis buffer and analyzed by immunoblotting to examine the expression of transfected proteins. Another aliquot was lysed in buffer A (6 M guanidine-HCl, 0.1 M Na_2_HPO_4_/NaH_2_PO_4_, 10 mM Tris-HCl, pH 8.0, 5 mM imidazole, and 10 mM β-mercaptoethanol) and subjected to sonication for a total of 60 s. Cell lysates were incubated with 40 µL pre-equilibrated Ni-NTA beads overnight at 4°C. The beads were washed six times sequentially with buffers A, B (8 M urea, 0.1 M Na_2_HPO_4_/NaH_2_PO_4_, 10 mM Tris-HCl, pH 8.0, and 10 mM β-mercaptoethanol), and C (same as B except pH 6.3), respectively. Beads with bound proteins were then boiled in a 2× SDS loading buffer with 200 mM imidazole and were subjected to immunoblotting.

### Statistical analysis

All experiments were performed independently at least three times. Statistical analyses were performed using an unpaired, two-tailed Student *t*-test. ^*^P < 0.05 was considered statistically significant; ^**^P < 0.01 was considered statistically highly significant.
